# Supplementary material for: Probing the origin of matching functional jaws: roles of Dlx5/6 in cranial neural crest cells
Source: Sci Rep. 2018 Oct 8;8:14975. doi: 10.1038/s41598-018-33207-2 (PMC6175850; doi:10.1038/s41598-018-33207-2)
Supplement: Supplementary file 1 — Supplementary Information [file 41598_2018_33207_MOESM1_ESM.doc]

**Probing the origin of matching functional jaws: roles of *Dlx5/6* in cranial neural crest cells.**

Miki Shimizu^1,+^, Nicolas Narboux-Nême^2,+^, Yorick Gitton^2,+^, Camille de Lombares^2^, Anastasia Fontaine^2^, Gladys Alfama^2^, Taro Kitazawa^1^, Yumiko Kawamura^1^, Eglantine Heude^2^, Lindsey Marshall^2^, Hiroki Higashiyama^1^, Youichiro Wada^3^, Yukiko Kurihara^1^, Hiroki Kurihara^1,4,*^ and Giovanni Levi^2,*^

^1^ Department of Physiological Chemistry and Metabolism, The University of Tokyo, 7-3-1 Hongo, Bunkyo-ku, Tokyo 113-0033, Japan.

^2^ Evolution des Régulations Endocriniennes, CNRS, UMR7221, Dept. AVIV, Muséum National d'Histoire Naturelle, Paris, France.

^3^ Isotope Science Center and Research Center for Advanced Science and Technology, The University of Tokyo, Tokyo 153-8904, Japan.

^4^ Core Research for Evolutional Science and Technology (CREST), Japan Science and Technology Agency (JST), Chiyoda-ku, Tokyo, 102-0076, Japan.

^+^ These authors contributed equally to the work

* Co-corresponding authors

**Supplementary Information**

**Supplementary Table 1.**


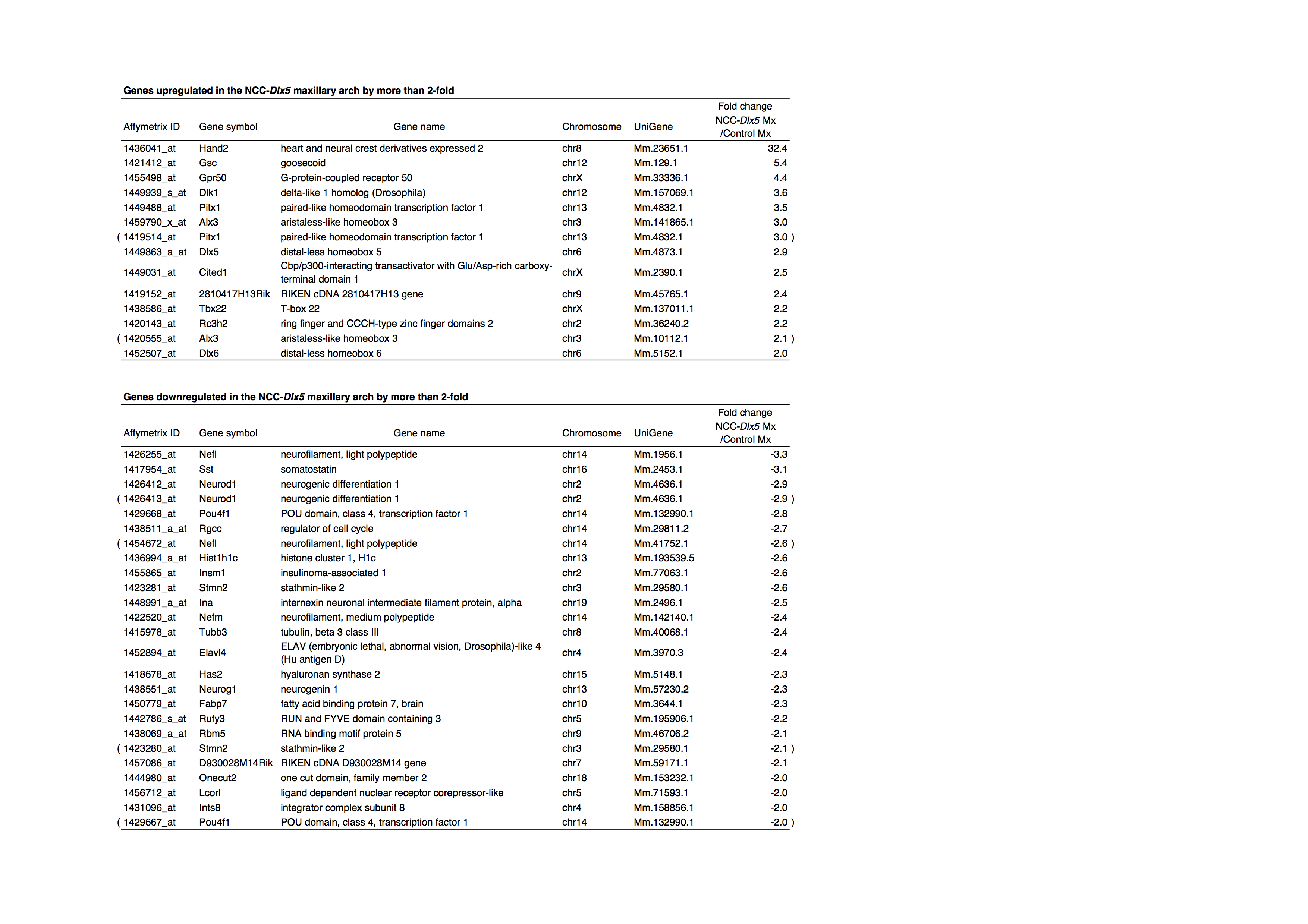
**Genes up- or down-regulated in the *NCCDlx5*  maxillary arch.**

**Supplementary Table 2.**

**Comparison of genes affected in the *NCC^Dlx5^*  maxillary arch with those affected in the *Dlx5/6*-null mandibular arch.**

**
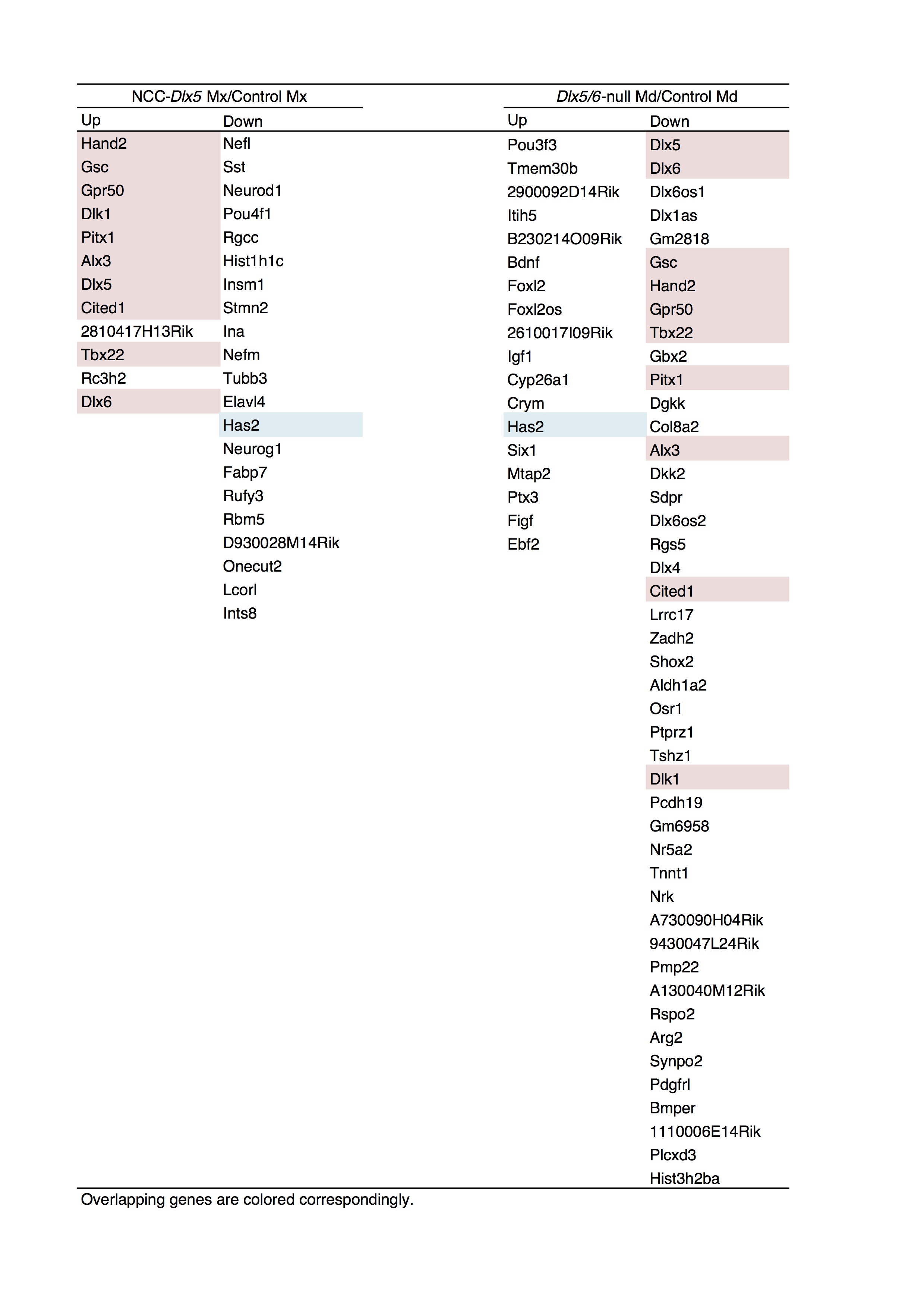
**

**Supplementary figures and legends:**

**Figs. Supplementary 1_4: 3D pdf files to be opened in Acrobat, which allows selection and manipulation of 3D reconstructed craniofacial structures described in this study.**

Colour code as in Figs. 3 and 4: Yellow, premaxillary bone; purple, maxillary bones; green, dentary bone; blue Meckelian cartilage; red, tongue; grey, teeth. Using the Acrobat functions different structures can be visualized or not, or shown with different options.

**Fig. S1:** Control craniofacial skeleton at E18.5

**Fig. S2:** *Dlx5/6^-/-^* craniofacial skeleton at E18.5

**Fig. S3:** *NCC^∆Dlx5/6^* craniofacial skeleton at E18.5

**Fig. S4:** *NCC^Dlx5^* craniofacial skeleton at E18.5

**Fig. S5: Morphological analysis of the transformation of the incus/malleus region in *NCC^Dlx5^* E18.5 foetuses.**

**
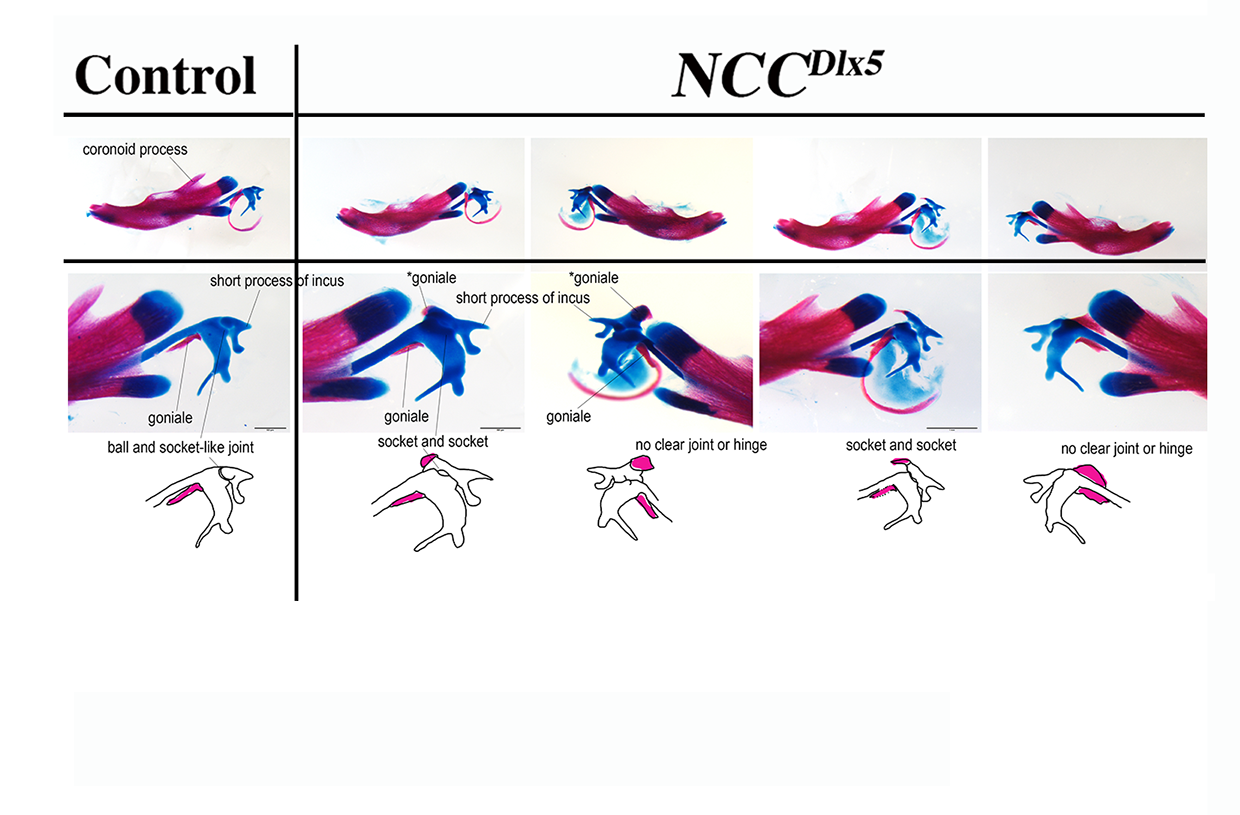
**

The elongation of the short process of the incus, the presence of a small ectopic bone, which could be interpreted as a duplicated gonial bone, adjacent to the incus, and the fact that the malleus-incus joint, which is normally of ball-and-socket type, is symmetric suggest a partial transformation of the incus in a malleus-like structure.
